# Identification of candidate genes for gelatinization temperature, gel consistency and pericarp color by GWAS in rice based on SLAF-sequencing

**DOI:** 10.1371/journal.pone.0196690

**Published:** 2018-05-10

**Authors:** Xinghai Yang, Xiuzhong Xia, Yu Zeng, Baoxuan Nong, Zongqiong Zhang, Yanyan Wu, Faqian Xiong, Yuexiong Zhang, Haifu Liang, Guofu Deng, Danting Li

**Affiliations:** 1 Rice Research Institute, Guangxi Academy of Agricultural Sciences, Nanning, Guangxi, China; 2 Biotechnology Research Institute, Guangxi Academy of Agricultural Sciences, Nanning, Guangxi, China; 3 Cash Crops Research Institute, Guangxi Academy of Agricultural Sciences, Nanning, Guangxi, China; National Cheng Kung University, TAIWAN

## Abstract

Rice is an important cereal in the world. The study of the genetic basis of important agronomic traits in rice landraces and identification of genes will facilitate the breed improvement. Gelatinization temperature (GT), gel consistency (GC) and pericarp color (PC) are important indices of rice cooking and eating quality evaluation and potential nutritional importance, which attract wide attentions in the application of genetic and breeding. To dissect the genetic basis of GT, GC and PC, a total of 419 rice landraces core germplasm collections consisting of 330 *indica* lines, 78 *japonica* lines and 11 uncertain varieties were planted, collected, then GT, GC, PC were measured for two years, and sequenced using specific-locus amplified fragment sequencing (SLAF-seq) technology. In this study, 261,385,070 clean reads and 56,768 polymorphic SLAF tags were obtained, which a total of 211,818 single nucleotide polymorphisms (SNPs) were discovered. With 208,993 SNPs meeting the criterion of minor allele frequency (MAF) > 0.05 and integrity> 0.5, the phylogenetic tree and population structure analysis were performed for all 419 rice landraces, and the whole panel mainly separated into six subpopulations based on population structure analysis. Genome-wide association study (GWAS) was carried out for the whole panel, *indica* subpanel and *japonica* subpanel with subset SNPs respectively. One quantitative trait locus (QTL) on chromosome 6 for GT was detected in the whole panel and *indica* subpanel, and one QTL associated with GC was located on chromosome 6 in the whole panel and *indica* subpanel. For the PC trait, 8 QTLs were detected in the whole panel on chromosome 1, 3, 4, 7, 8, 10 and 11, and 7 QTLs in the *indica* subpanel on chromosome 3, 4, 7, 8, 10 and 11. For the three traits, no QTL was detected in *japonica* subpanel, probably because of the polymorphism repartition between the subpanel, or small population size of *japonica* subpanel. This paper provides new gene resources and insights into the molecular mechanisms of important agricultural trait of rice phenotypic variation and genetic improvement of rice quality variety breeding.

## Introduction

Rice is one of the most important food crops in the world [1].With the increasing standard of living of the people in China, the cooking and eating, and nutritional quality has attracted more attention. GT and GC are important factors affecting cooking and eating quality of rice. Anthocyanin or proanthocyanidin accumulated in the pericarp of rice are bioactive flavonoids [2], which have strong antioxidant and anti-mutagenic functions as plant nutrients, and play an important role in human health [3], so black rice or red rice are also more and more popular among consumers [4].

So far, many researches have reported the genetic basis of GT, GC and PC. It was concluded that *ALK* is the major effect gene for GT [5,6] and *Wx* has a minor effect on GT [7]. GC is an eating and cooking quality related trait with complicated genetic basis, and more than 20 QTLs for GC have been detected (http://www.gramene.org/) in rice, which located on chromosome 1, 2, 4, 6, 7, 11. Anthocyanin or proanthocyanidin biosynthesis pathway is a very complex process in plant [8], and molecular mechanism of pericarp anthocyanin and proanthocyanidin biosynthesis in rice is still unclear [9].

The increased rice yield, improved quality and advanced resistance to biotic and abiotic stress play an important role in solving the world food problem, improving people’s life quality and reducing environmental pollution. Identification and utilization of favorable genes in rice germplasm resources is the foundation of rice breeding. Guangxi is likely the origin of cultivated rice [10], which possesses a large number of rice landraces containing rich natural variation and genetic diversity, and it is the important genetic resources for breed improvement.

Quantitative trait loci (QTLs) mapping has been widely used to explore the genetic basis of complex agronomic traits in different crops. However, almost studies need to construct mapping populations, which is very time-consuming and painstaking [11]. In recent years, with the rapid development of high-throughput sequencing technology and reduced sequencing cost, genome-wide association study (GWAS) based on SNPs has become a new method for studying important agronomic traits in rice. Huang et al. [12] detected 37 SNPs significantly associated with 14 agronomic traits by GWAS with 517 Chinese rice landraces, which per locus explained 36% of the phenotypic variation on average and 7 loci were consistent with the results of previous studies. Subsequently, Huang et al. [13] used 950 worldwide rice germplasm collections to carry out GWAS analysis on flowering and grain yield traits, and confirmed 18 candidate genes. Chen et al. [14] associated 36 candidate genes with 840 metabolites by GWAS analysis with 529 rice germplasm collections. Si et al.[15] identified *OsSPL13* affecting the grain size by GWAS and elucidated the molecular mechanism. Yano et al. [16] obtained 26 significant loci associated with heading stage by GWAS, two of which were located on *Hd2* and *Hd6* reported previously. Meyer et al. [17] confirmed 28 salt tolerance related loci located on 11 genes regions with 93 african cultivated rice collections by GWAS. Li et al. [18] identified a broad-spectrum blast resistance related gene *Bsr-d1* through GWAS. These results showed that the excellent alleles in rice germplasm could be identified efficiently and quickly using GWAS technology.

In this study, we used a core collection of 419 rice landraces from Guangxi to dissect the molecular basis of GT, GC and PC, the whole panel was genotyped based on SLAF-seq technology, the three traits were measured for twice during 2014 and 2015, and then the GWAS was performed. The research provided genetic resources for molecular breeding and shed light on rice quality improvement.

## Materials and methods

### Plant materials

The whole panel were composed of a core collection of 419 landraces collected from rice germplasm from Guangxi (China, 20.90–26.40 °N, 104.43–112.07 °E). The lines were planted in Nanning experimental field (China, 22.85°N, 108.26°E) from July 2014 to November 2014 and from July 2015 to November 2015, in a randomized block design with two replications within each year. In each replicate, three rows and ten plants per row for each landrace was planted with 20 cm×13.2 cm spacing. The soil in the experimental station is of weak acidity with pH = 6.48, containing 0.12% total phosphorus, 0.11% total phosphorus, 1.78% total potassium, fertilizer (N-P_2_O_5_-K_2_O) was applied at the rate of 150-75-135 kg/ha. For each landrace, five randomly chosen plants were harvested when they were mature and rice grains were air-dried until the moisture content reached 13% and then dehulled to obtain brown rice. Each brown rice sample was milled by a polishing machine. After the rice was milled, the chaff and the chips in samples were removed using a 1.5 mm diameter sifter.

### Determination of GT

GT was determined using the alkali digestion test [19]. Six grains of mature and polished rice were placed into a box (5cm×5cm×2cm) containing 10 ml 1.7% NaOH solution, and the box was laid into 30 °C ± 2 °C incubator for approximately 23 h. The formula for computing ADV is as follows:

$$ADV=\frac{\sum \left(G\times N\right)}{6}$$

The ADV refers to the alkali digestibility value, G is the grade of every grain of rice; N is the grain number of same grade. The grade of alkali digestibility is that 1, 2, 3, 4, 5, 6, 7 refer to grain unchanged, grain expansion, grain expansion and ring incomplete or narrow, grain enlargement and ring intact and wide, grain cracking and ring intact and wide, grain partially dispersed and dissolved and fused with the ring, and grain completely dispersed respectively. A set of standard samples of known GT including high, medium and low classes were used as control. The protocol was repeated two times, and the difference between two replicates was less than 0.5.

### Determination of GC

GC was measured in millimeters according to the method of Cagampang et al. [20]. 100 mg of rice flour was placed into tube, then 0.2 ml 0.025% thymol blue solution was added. After fully dispersed, adding 2 ml 0.2 mol/L NaOH solution and putting the tube into the boiling water for heating 8 min, cooling the tube in 0 °C cold water for 20 min, and then measuring the flow length of the rice gum. The protocol was repeated twice, and the difference of two results was less than 7mm.

### Determination of PC

GC was measured according to the method of Han et al.[21] and Huang et al. [12]. Selecting 20 grains of mature and unpolished rice randomly, and comparing the color with the control standard swatches. If the color of the seed coat is not consistent, the variation is calculated.

### SLAF sequencing and SNP genotyping

Total genomic DNA was extracted from young quadrifoliate leaves of all rice landraces using cetyltrimethyl annonium bromide (CTAB) protocol [22], and digested by two restriction enzymes RsaI and HaeIII. The SLAF sequencing was performed on an IlluminaHiseq 2500 system. The polymorphic SLAF tags were obtained by clustering the clean reads using BLAT software [23], aligned to reference genome (*Oryza sativa* L. *japonica* cv. Nipponbare, http://plants.ensembl.org/Oryza_sativa/Info/Index) using the BWA software [24], and then the SNP calling was performed using GATK [25] and SAMtools packages [26]. A total of 208,993 SNPs with a minor allele frequency (MAF)>0.05 and integrity > 0.5 was retained for GWAS.

### Genetic kinship calculation, phylogenetic tree construction, principal component analysis, population sturcture

Based on the 208,993 high quality SNPs, the phylogenetic tree was constructed by MEGA5 [27]. The pairwise kinship was carried out using the SPAGeDi software package [28], and the population structure was analysed by ADMIXTURE software [29], which the subpanel number was predicted from 1 to 10. The kinship and population structure analysis was performed for the whole panel, *indica* subpanel and *japonica* subpanel in convenience of subsequent GWAS for the three combinations, respectively. Principal component analysis (PCA) was performed using GAPIT [30].

### Genome-wide association study

In this study, in order to eliminate the effort of population structure between *indica* subpopulation and *japonica* subpopulation, GWAS was performed for the whole panel, *indica* subpanel and *japonica* subpanel respectively using the mixed linear model (MLM) of TASSEL V3 [31], which took the population structure and kinship into consideration, and the significant *P* value was set to 4.79×10^−8^.

## Result

### Phenotypic variation of GT, GC and PC

The alkali spreading value can be used to measure the GT, which is inversely related to GT, and ranged from 0 to 6.15 with an average of 2.86 in 2014, and 0 to 7 with an average of 3.74 in 2015; The GC spanned 26 to 100 with an average of 67.91 in 2014, and 6 to 100 with an average of 70.94 in 2015; for the PC trait, the number of rice landraces with white, red, black color is 308, 97, 14 respectively (S1 Table).

### Analysis of SLAF-seq data and development of SNPs

After sequencing data quality control, a total of 67,665 SLAF tags were obtained with an average sequencing depth of 8.75×. Ultimately, 56,768 polymorphic SLAF tags were retained when aligned to the reference genome (S2 Table).

**Fig 1:**
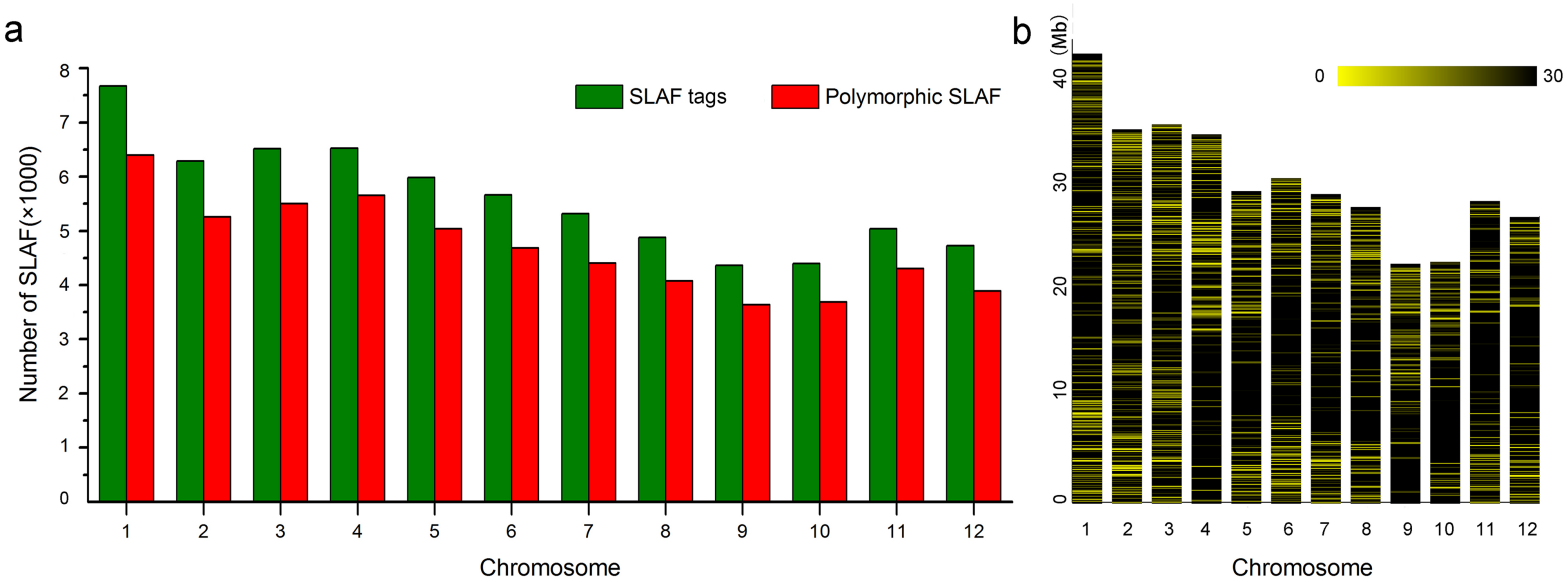
Distribution of SLAF tags and SNPs on chromosomes of 419 rice landraces. (a) SLAF tags on chromosomes; (b) SNPs on chromosomes.

A total of 211,818 SNPs were identified using the GATK and SAMtools software package, of these, the number of SNPs with MAF > 0.05 and integrity> 0.5 was 208,993, which distributed on every chromosome with a mean number 17,416 per chromosome. For the high quality SNPs, 104,068 SNPs located in gene region, and 107750 SNPs in intergenic region. The chromosome 10 possessed the most SNP density while chromosome 2 for the fewest SNP density (Fig 1, S3 Table).

### Genetic kinship calculation, phylogenetic tree construction and principal component analysis

**Fig 2:**
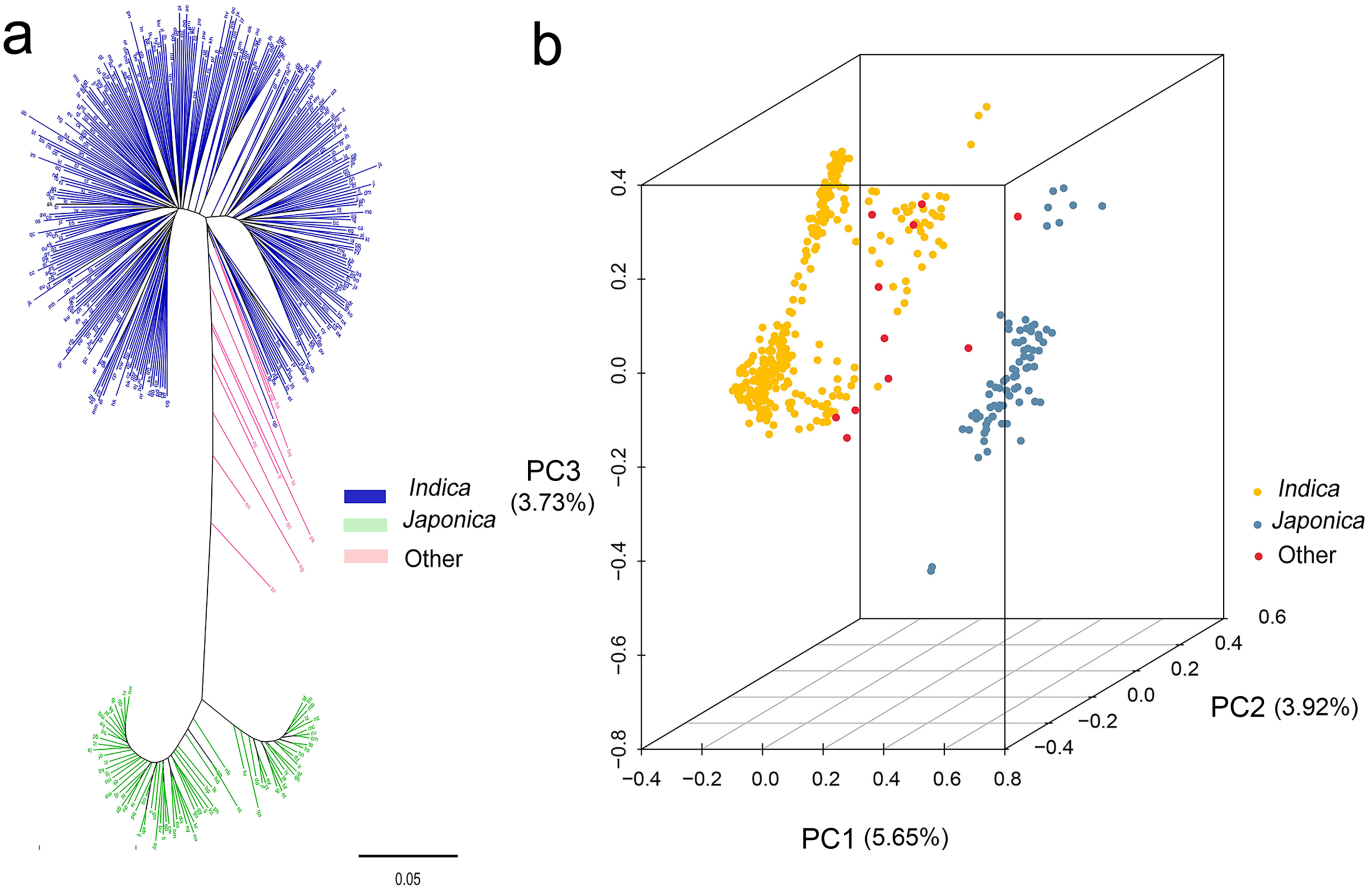
Phylogenetic tree and PCA analysis for 419 rice landraces. (a) Clustering of 419 rice landraces; (b) PCA analysis of 419 rice landraces.

Based on the 208,993 high quality SNPs, 87,571 pairwise calculations for all 419 rice landraces were carried out. Of these, the number of pairs reached up to 45,709, which the genetic relationship coefficients <0.05. The phylogenetic tree was clustered two mainly panels in accordance with the *indica* and *japonica* subpanels. The first two principal components explained 5.65% and 3.92% of the genetic variation, respectively (Fig 2).

### Analysis of population sturcture and GWAS

**Fig 3:**
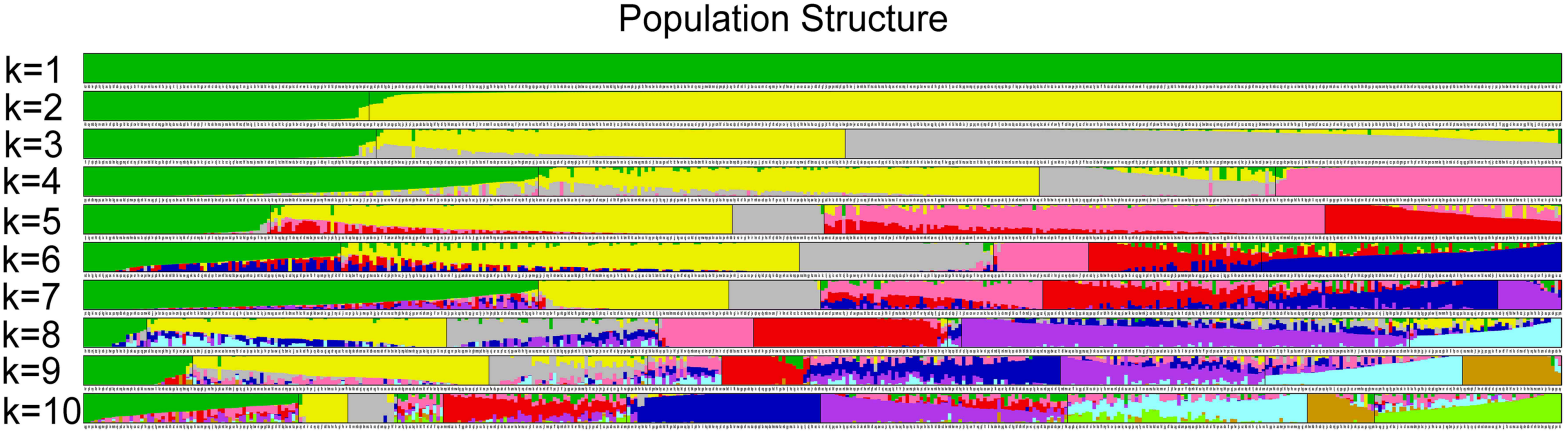
Population structure of all 419 rice landraces.

Based on the error rate of 5-fold cross-validation, the ancestor number was confirmed to 6 for all the 419 rice landraces. The six subpanels respectively contain 330 *indica* varieties, 78 *japonica* varieties (Fig 3).

### GWAS mapping in rice landraces

**Fig 4:**
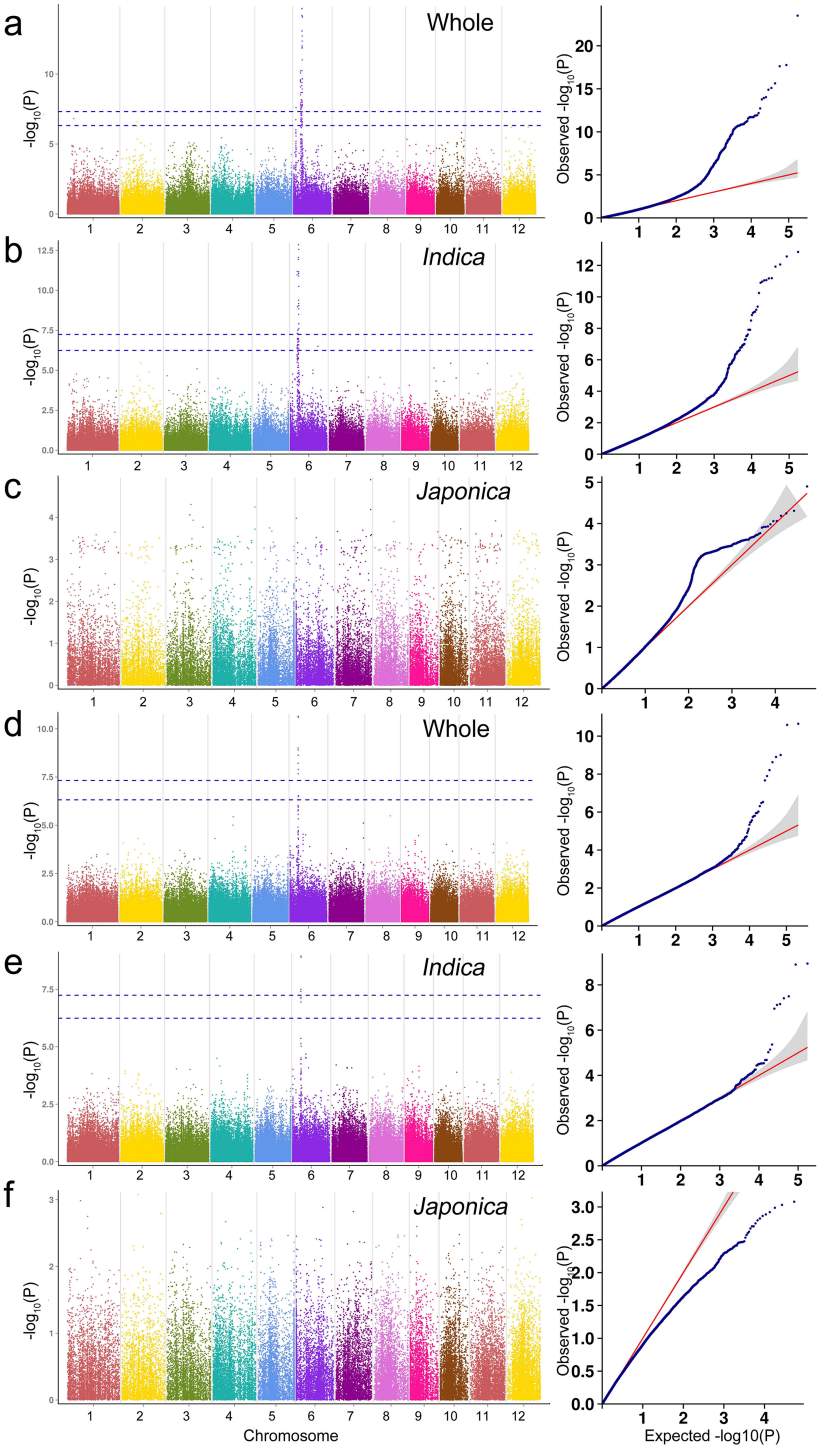
Genome-wide association studies for gelatinization temperature based on MLM model. (a-c) Manhattan plots and quantile-quantile plots for the whole panel and the two subpanels in 2014; (d-f) Manhattan plots and quantile-quantile plots for the whole panel and the two subpanels in 2015.

**Fig 5:**
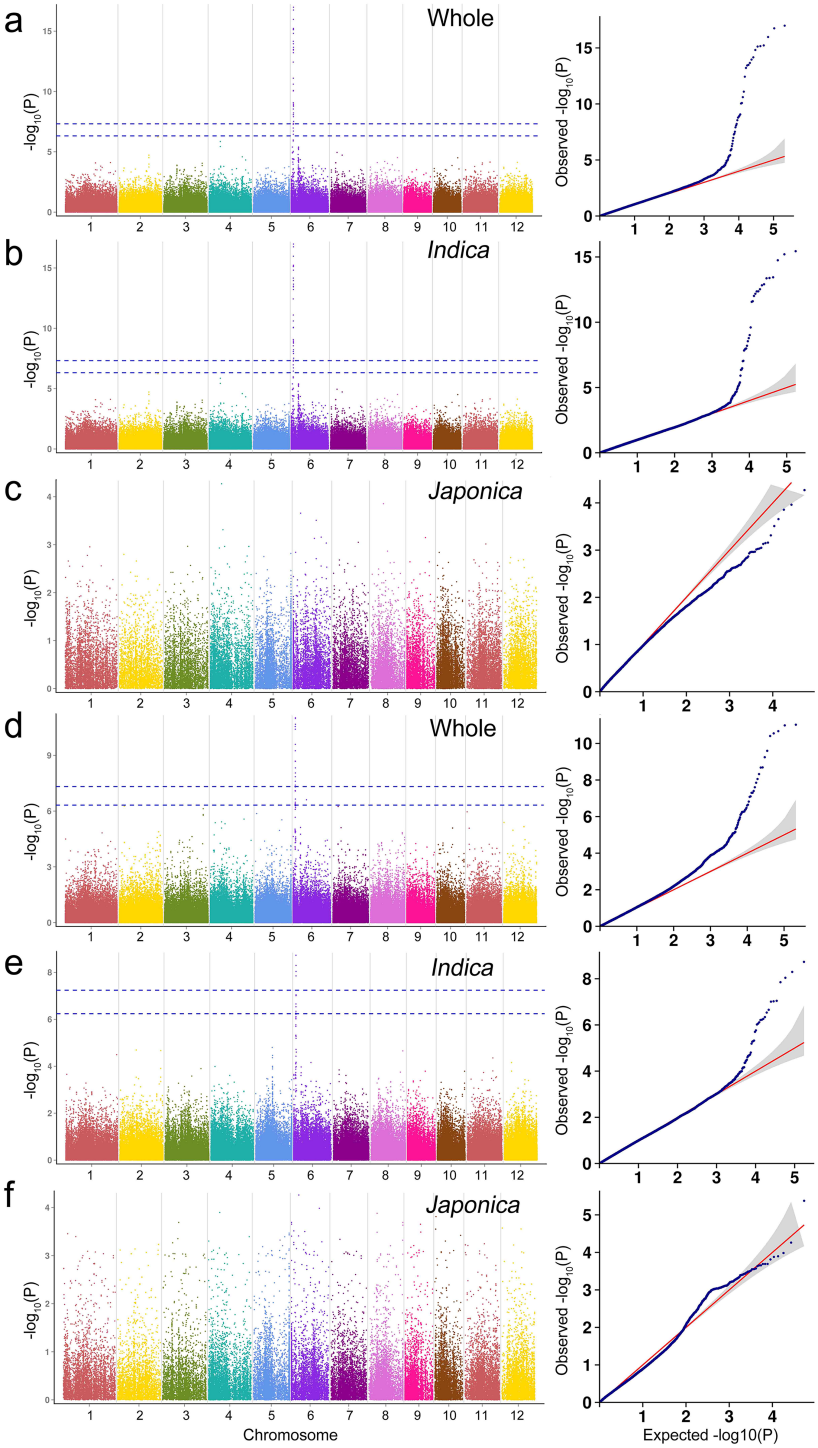
Genome-wide association studies for gel consistency based on MLM model. (a-c) Manhattan plots and quantile-quantile plots for the whole panel and the two subpanels in 2014; (d-f) Manhattan plots and quantile-quantile plots for the whole panel and the two subpanels in 2015.

**Fig 6:**
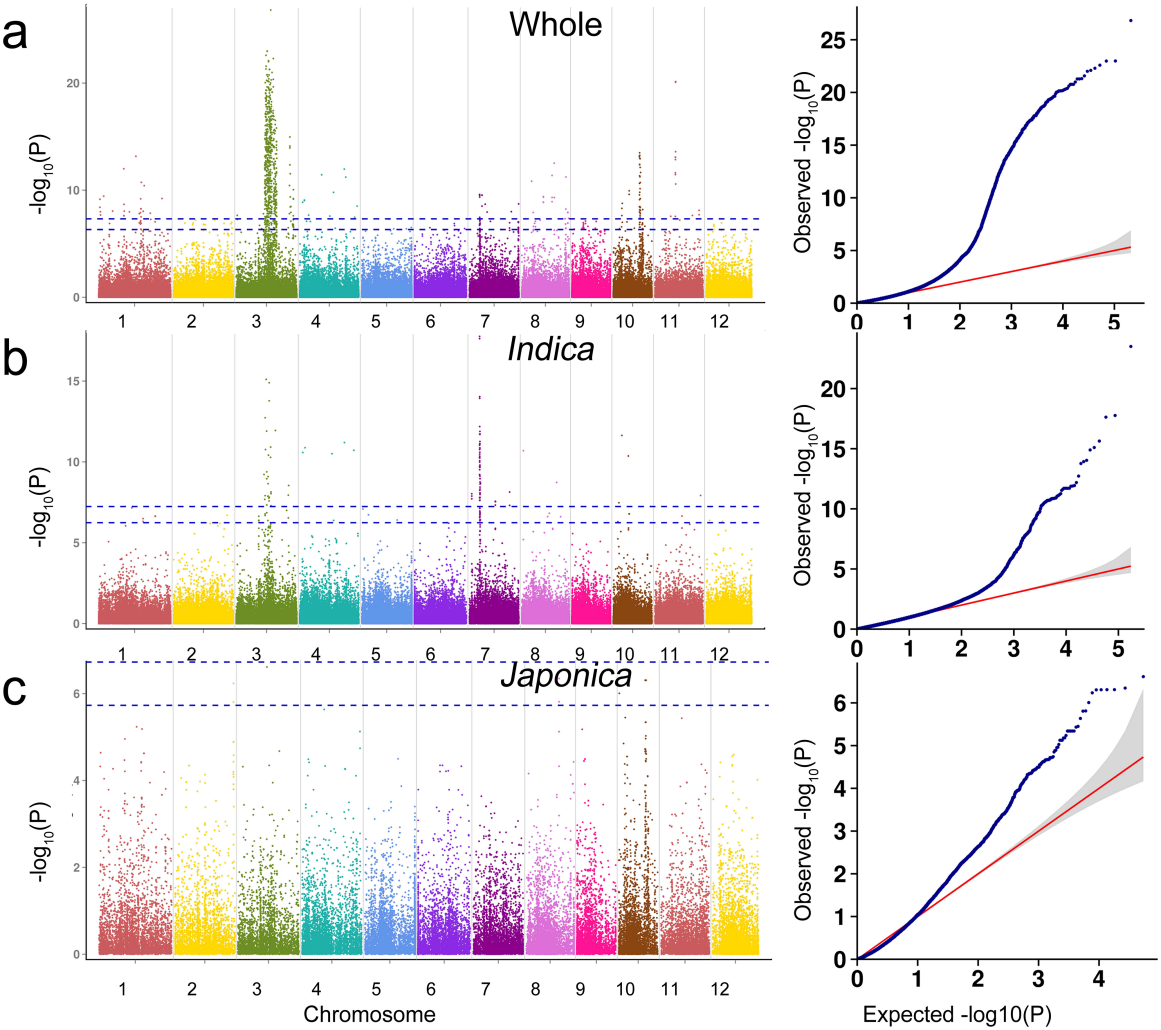
Genome-wide association studies for pericarp color based on MLM model. (a-c) Manhattan plots and quantile-quantile plots for the whole panel and the two subpanels.

The whole panel composed of 419 rice landraces, *indica* subpanel (330 rice landraces) and *japonica* subpanel (78 rice landraces) were used for GWAS respectively in order to avoid population structure noise. Only one QTL was detected for GT, but in both the whole panel and *indica* subpanel. Similarly, only one association with GC was obtained, also both the whole panel and *indica* subpanel. Interesting, the two QTLs were both located on chromosome 6 (Figs 4 and 5). For the PC trait, 8 QTLs were detected in the whole panel on chromosome 1, 3,4,7, 8, 10 and 11, and 7 QTLs in the *indica* subpanel on chromosome 3, 4, 7, 8, 10 and 11 (Fig 6).

### GWAS on GT

GT is one of the most important indexes to evaluate the cooking and eating quality of rice. The GWAS for GT was conducted for the whole panel, *indica* subpanel and *japonica* subpanel successively. In 2014, for the whole panel of 419 rice lanraces, GWAS detected a total of 48 GT related SNPs, the 26 SNPs of which can also be detected in *indica* subpanel, and the significant SNPs distributed on the 1,807,797 bp-7,174,281 bp of chromosome 6 (Fig 4, S4 Table). In the whole and *indica* panels, the most significant SNPs for GT were Chr6_6733351 (*P* = 2.04×10^−15^) and Chr6_6740370 (*P* = 1.39×10^−13^). In 2015, 8 GT related SNPs were identified in the whole panel, of which 4 SNPs can also be detected in *indica* panel, and the positions of these SNPs ranged from 6,740,370 bp to 6,927,719 bp on chromosome 6 (Fig 4, S4 Table). However, no significant SNP was detected for the *japonica* subpanel in the both 2014 and 2015 year. In the whole panel and *indica* subpanel, the most significant SNPs were both Chr6_6879531 (*P* = 2.23×10^−11^; *P* = 1.14×10^−9^). Chr6_6733351 and Chr6_6740370 were located in 15 kb and 8 kb upstream of the *ALK* gene, respectively (Table 1).

**Table 1:** A sbuset of associated loci and candidate genes.

Panel	Trait	Chromosome	Position	*P*-value	Cadidate gene	Annotation
**Full**	GT	6	6733351	2.04E-15	*LOC_Os06g12450*	Soluble starch synthase 2–3
**Indica**	GT	6	6740370	1.39E-13	*LOC_Os06g12450*	Soluble starch synthase 2–3
**Full**	GC	6	1797551	1.03E-17	*LOC_Os06g04200*	Starch synthase
**Indica**	GC	6	1797551	3.62E-16	*LOC_Os06g04200*	Starch synthase
**Full**	PC	1	22408336	6.8E-14	*LOC_Os01g44260*	Dihydroflavonol-4-reductase
**Full**	PC	3	20743207	1.5E-27	*LOC_Os03g38210*	MYB family transcription factor
**Indica**	PC	3	17963359	7.88E-16	*LOC_Os03g31230*	MYB family transcription factor
**Full**	PC	3	32304963	1.07E-15	*LOC_Os03g60509*	Chalcone isomerase
**Indica**	PC	3	31521682	2.89E-09	*LOC_Os03g60509*	Chalcone isomerase
**Full**	PC	4	26803164	1.07E-12	*LOC_Os04g47059*	bHLH transcription factor
**Indica**	PC	4	26803164	6.35E-11	*LOC_Os04g47059*	bHLH transcription factor
**Full**	PC	7	6069266	2.52E-10	*LOC_Os07g11020*	bHLH transcription factor regulating proanthocyanidin production in seeds
**Indica**	PC	7	6069266	1.72E-18	*LOC_Os07g11020*	bHLH transcription factor regulating proanthocyanidin production in seeds
**Full**	PC	8	12968543	3.96E-10	*LOC_Os08g21660*	WD domain, G-beta repeat domain containing protein
**Indica**	PC	8	938782	2.02E-11	*LOC_Os08g21660*	WD domain, G-beta repeat domain containing protein
**Full**	PC	10	15835327	3.25E-14	*LOC_Os10g30690*;*LOC_Os10g30719*	MYB family transcription factor
**Indica**	PC	10	5131361	2.32E-12	*LOC_Os10g30690*;*LOC_Os10g30719*	MYB family transcription factor
**Full**	PC	11	12447381	8.27E-21	*LOC_Os11g45740*	MYB family transcription factor
**Indica**	PC	11	27509828	1.19E-08	*LOC_Os11g45740*	MYB family transcription factor

The *ALK* gene encodes soluble starch synthaseIIand is responsible for GT of rice [32]. Some researchers have analyzed the SNP in *ALK* gene, and considered that SNP variation was the main factor of GT change [33–35]. We also found a SNP Chr6_1807797 (*P* = 2.53×10^−8^) which was significantly associated with GT located in the *Wx* gene region in 2014, which further verified the correlation between the *ALK* and the *Wx* gene (Fig 4).

### GWAS on GC

GC is a complex quality trait in rice, and views about the genetic basis of GC were not consistent among different researchers. GC is inversely related to amylose content, we performed pearson correlation analysis for GC and amylose content trait, and reached the same conclusion (S5 Table). Many researchers believed that the *Wx* gene located on the Chromosome 6 was the major gene controlling GC [36–38], and some other GC-related QTLs located on chromosome 1, 2, 4, 6, and 7, 11 were also detected [39,40]. In 2014, in the whole panel and *indica* subpanel, GWAS detected a total of 28 and 24 significant SNPs respectively, the QTL located in the interval of 1,607,061 bp-1,958,767 bp on chromosome 6, and the most significant SNPs were both Chr6_1797551 (*P* = 1.03×10^−17^; *P* = 3.62×10^−16^) for both whole and *indica* panels (Fig 5, S6 Table). In 2015, 13 and 4 significant SNPs were confirmed for the whole and *indica* panels respectively. These SNPs sited in 1,661,801 bp-1,822,395 bp of chromosome 6 (Fig 5, S6 Table). However, no SNP was detected for the *japonica* subpanel in both 2014 and 2015 years. For the whole and *indica* panels, the most significant SNPs were Chr6_1807797 (*P* = 9.48×10^−12^) and Chr6_1754453 (*P* = 1.86×10^−9^) respectively. The Chr6_1754453, Chr6_1797551 and Chr6_1807797 were located 11.2 kb upstream, 26.9 kb and 37.2 kb downstream of the *Wx* gene, respectively (Table 1).

### GWAS on PC

PC is an important nutritional quality trait of rice. For the whole and *indica* panels, 763 and 99 significant SNPs were identified respectively, however no significant SNPs were detected for *japonica* panel (Fig 6, S7 Table). For chromosome 1, 25 significant SNPs were detected in the whole panel, but no SNPs in *indica* subpanel. The most significant SNP was Chr1_22408336 (*P* = 6.80×10^−14^), located in the 2.97 Mb downstream of proanthocyanidins biosynthesis gene *Rd* [41]. For chromosome 3, 647 and 27 significant SNPs were detected for the whole and *indica* pane respectively, which sited in two clearly divided QTLs. In the 17,126,203 bp-24,432,074 bp, the most significant SNP was Chr3_20743207 (*P* = 1.50×10^−27^) and Chr3_17963359 (*P* = 7.88×10^−16^) for the whole and *indica* panels respectively. The *LOC_Os03g38210* and *LOC_Os03g31230*, which both encode the MYB family transcription factors, were located in the 459.6 kb downstream of Chr3_20743207 and 179.2 kb upstream of Chr3_17963359 respectively (Table 1). In the 31,984,778 bp-34,408,499 bp of chromosome 3, for the whole and *indica* panels, the most significant SNPs were Chr3_32304963 (*P* = 1.07×10^−15^) and Chr3_31521682 (*P* = 2.89×10^−9^); the Chr3_31521682 and Chr3_32304963 located in 2.9 Mb and 2.1 Mb upstream of *OsCHI*, which encode chalcone isomerase [42]. For chromosome 4, 11 and 5 significant SNPs were found between whole and *indica* panels, and the most significant SNPs were both Chr4_26803164 (*P* = 1.07×10^−12^, *P* = 6.35×10^−12^). *Kala4* was located in the 1.11 Mb downstream of Chr4_26803164, which encodes a bHLH transcription factor for anthocyanins biosynthesis in black rice [2]. For chromosome 7, 12 significant SNPs were detected in the whole panel, and 61 significant SNPs in *indica* subpanel. The most significant SNP was Chr6_6069266 (*P* = 2.52×10^−10^; *P* = 1.72×10^−18^), located in the *Rc* gene region (Table 1), which encodes bHLH transcription factor regulating proanthocyanidin production in pericarp [43]. For chromosome 8, 17 significant SNPs for the whole panel and 2 SNPs for *indica* subpanel were detected. For the whole panel, the *LOC_Os08g21660* encoding a WD domain, G-beta repeat domain contained protein located in 59.9 kb upstream of Chr8_12968543 (*P* = 3.96×10^−10^) (Table 1), which also located in the QTL ranging from 938,782 bp to 20,998,896 bp of *indica* subpanel. For chromosome 10, 37 significant SNPs for the whole panel and 3 significant SNPs for *indica* panel were found, and there was no identical SNPs between them. Forthe whole panel, *LOC_Os10g30690* and *LOC_Os10g30719* (Table 1), which encode MYB family transcription factor, were located in 148.5 kb and 162.3 kb downstream of the most significant SNP Chr10_15835327 (*P* = 3.25×10^−14^) respectively. For Chromosome 11, 14 and 1 significant SNPs were detected for the whole and *indica* panels respectively. In this region, we found a candidate gen*e*, *LOC_Os11g45740*, which encodes MYB family transcription factor.

## Discussion

Rice is the most important staple crop worldwide, and feeding a fast growing population, so it is emergent to identify genes related to agronomically important traits. Until now, yield [44–47], quality [32,48,49], and resistance genes/QTLs [50–52] have been identified and cloned through biparental linkage mapping in rice. However, QTL analysis is very time-consuming and painstaking [11]. Recently, previous studies have documented that GWAS could be a useful tool to dissect the genetic changes for complicated traits has some advantages [49,50]. First, GWAS can efficiently detect multiple QTLs in the same population meanwhile; Second, the associated population includes the majority of variations of the related loci. Therefore, the GWAS not only can help us understand the gene function, but also discover the favorable alleles for genetic improvement of plants. In humans, even with extremely low-coverage sequencing and imputation increases power for GWAS [53]. Wang et al. [54] had also documented that low-coverage whole-genome sequencing is an effective strategy for GWAS in rice. In this study, in order to dissect the genetic basis of GT, GC and PC, we performed GWAS based on SLAF-seq technology [55]. The results also confirmed the conclusion that the SLAF-technology could effectively and accurately identify the associated genes, though the obtained genome information is less than information, which is obtained by whole genome resequencing.

Core collection is a subset of the germplasm resources, representative of the genetic diversity and geographical distribution of the entire population with the minimum number of genetic resources [56]. Guangxi province of China is likely to be the origin of cultivated rice [10], which possesses a large number of rice germplasms and genetic resources. In this study, a GWAS for GT, GC and PC of 419 rice landraces core germplasms from Guangxi was performed. The GWAS results in 2014 and 2015 showed that the QTLs of GT, GC and PC were relatively stable in different environmental conditions.

GT, an important index for evaluating rice cooking quality, is controlled by a major gene *ALK* confirmed by previous researches [6,32,38,57]. Tian et al. [7] showed that the *Wx* gene has a minor effect on GT. Recently, Wang et al. [58] identified GT related loci *qGT3*, *qGT6*, *qGT7*, which *ALK* is the candidate gene of *qGT6*. In this study, one QTL on chromosome 6 was detected in the whole panel (Chr6_6733351, P = 2.04×10^−15^) and *indica* subpanel (Chr6_6740370, P = 1.39×10^−13^) which corresponds to *ALK* (6748358–6753338 bp) (Table 1), a confirmed major gene for GT [32].

GC is a relatively complex quality trait, so the QTLs of GC ate not consistent. So far, more than 20 GC related QTLs are included in the Gramene database (http://www.gramene.org/), which locate on chromosome 1,2, 4, 6, 7, 11. Tan et al. [6], Bao et al. [59] and Su et al. [36] asserted that *Wx* is the major gene for GC. Li et al. [39] found two QTLs, *gc2*.*1* and *gc7*.*1*, for GC on chromosome 2, 7. Gao et al. [38] confirmed that *ALK* is modifier gene for GC in rice. Tran et al. [37] found that a single nucleotide polymorphism in the *Wx* gene explains a significant component of GC. Swamy et al. [60] identified 6 QTLs for GC, *gc1*.*1*, *gc1*.*2*, *gc2*.*1*, *gc4*.*1*, *gc11*.*1*, located on chromosome 1, 2, 4, 11, which the position of *gc2*.*1* is near to that of *gc2*.*1* identified by Li et al. [39]. Based on RAD-seq technology, Peng et al. [40] detected a QTL *qGC6-1*, and this QTL corresponded to the *Wx* encoding GBSSI, which is the major gene controlling amylose synthesis [7,61,62]. Wang et al. identified [58] *qGC2*, *qGC4*, *qGC6*, *qGC11*, *qGC12* affecting GC located on chromosome 2, 4, 6, 11, 12, which *qGC6* overlaps with the *Wx* gene. In our study, the highest peak on chromosome 6 in the whole panel (Chr6_1797551, P = 1.03×10^−17^) and *indica* subpanel (Chr6_1797551, P = 3.62×10^−16^), was located close to *Wx* gene [35,40, 61,62].

The accumulation of anthocyanins and proanthocyanidins in the pericarp leads to red and black pericarp rice respectively. The anthocyanin or proanthocyanidins metabolism pathway in various plants has been explored to some extent, which is not yet fully understood in rice. So far, some structural genes involved in anthocyanin biosynthesis has been identified, such as *OsCHS*1 [63], *OsCHS2* [64], *OsCHI* [42], *OsF3H* [65], *OsF3’H* [64], *OsDFR* (*Rd*) [41] and *OsANS* [64]. About the regulatory genes, Furukawa et al. [43] cloned *Rc* gene located on chromosome 7, which encodes bHLH domain contained transcription factor and participates in the proanthocyanidin synthesis, which is consistent with our result. Oikawa et al. [2] identified *Kala4* controlling anthocyanin biosynthesis, identical to *OSB2* confirmed by Sakamoto et al. [66] in rice. Chin et al. [67] showed that the *OsC1* of chromosome 6 is related to the purple sheath in rice, encoding a MYB transcription factor. Recently, Sun et al. [68] put forward a *C*-*S*-*A* gene model controlling rice hull color, which C,S and A refer to *OSC1*, *Kala4* and *Rd* respectively, responsible for corresponding color production, tissue specific coloring and anthocyanin synthesis. Although anthocyanin synthesis related genes have been identified in rice, the key genes of anthocyanin synthesis in rice pericarp, such as MYB and WDR transcription factors, have not been reported. However, because of the relatively narrow genetic diversity of collections used for genetic mapping, these currently identified genes can not completely explain the pathway of anthocyanin or proanthocyanidins metabolism in rice. In theory, many genes that regulate the synthesis of anthocyanins or proanthocyanidins in rice are still not found. In this study, eight QTLs of PC were detected for the whole panel. The most significant SNP (Chr1_22408336, P = 6.80×10^−14^) on chromosome 1 was adjacent to the *Rd* participating in the anthocyanins and proanthocyanidins biosynthesis [41]. The *LOC_Os03g38210*, wich was the candidate gene for the most significant SNP on chromosome 3 encodes a MYB transcription factor, which are different locus of *Kala3* participating in anthocyanin biosynthesis for black rice [69]. In another significantly associated region (31,9847,78 bp -34,408,499 bp) on chromosome 3, among the genes forming the seventh significant SNP (Chr3_ 34389605, *P* = 9.17×10^−11^), *LOC_Os03g60509* encodes chalcone isomerase which is an important structural gene in the anthocyanins biosynthesis [42]. The anthocyanin biosynthesis key gene *Kala4* near the most significant SNP on chromosome 4 [2]. The *LOC_Os08g21660* locates in 54.4 kb upstream the most significant on chromosome 8 (Chr8_12968543, *P* = 3.96×10^−10^), and encodes a WD domain and G-beta repeat domain contained protein. So far, the WD40 domain contained gene has not been identified in rice, which is involved in the anthocyanin biosynthesis [2]. There are two genes (*LOC_Os10g30690* and *LOC_Os10g30719)* located in 148.5 kb and 162.3 kb downstream of the highest (Chr10_15835327, *P* = 3.25×10^−14^) on Chromosome 10 respectively, both encoding MYB transcription factor, which are not overlapped the *qPc10* identified by Wang et al. (2016) [54]. Compared to the whole panel, we did not detect loci associated with PC on chromosome 1 in the indica subpanel. On the chromosome 3, the most significantly associated locus was Chr_17963359 (*P* = 7.88×10^−16^) and the candidate gene was *LOC_Os03g31230* in the indica subpanel.

These results demonstrated that GWAS was a powerful tool for detecting favorable alleles. But no QTL was detected in *japonica* subpanel for the three traits and some significant SNPs have large physical distance from candidate genes, probably because of the polymorphism repartition between the subpanel [70], or small population size of *japonica* subpanel [71]. However, GWAS has some limitations: (i) the large effect variations and minor effect genes can not be identified easily by GWAS [71]; (ii) genetic heterogeneity can reduce the efficacy of mutation detection [71,72]; (iii) the population structure lead to false positive associations between phenotype and unlinked markers [16]; (iv) GWAS can be limited by the genetic characteristics of different species, for example, the LD decay of rice is lower than that of outcrossing maize [73], so the GWAS still can not replace the traditional map based clone to achieve fine mapping of the target gene [16,74].

## Conclusions

With the improvement of people’s living standard, the rice of high quality is more and more needed. The discovery and utilization of excellent germplasm can accelerate rice nutritional quality breeding. Based on SLAF-seq technology, GWAS for GT, GC and PC of 419 rice core collections from Guangxi was conducted, and associated genes, especially the anthocyanin or proanthocyanidin biosynthesis related genes on chromosome 3,8,10 and 11 were reported for the first time. This study shed light on the genetic analysis for important agricultural trait of rice and beneficial to plant breeder.

## Supporting information

S1 Table The alkali value, gel consistency and pericarp color of 419 rice landraces. For pericarp color, 1, 2 and 5 represent white, red and black pericarp, respectively. ‘-’ represents missing data.
(XLSX)

S2 Table Distribution on chromosomes of SLAF tags and polymorphic SLAF tags. (XLSX)

S3 Table SNPs distribution on chromosome. (XLSX)

S4 Table The significant SNPs of gelatinization temperature. (XLSX)

S5 Table The correlation coefficient of amylose content, gel consistency, gelatinization temperature in 2014 and 2015. * and ** indicate 0.05 and 0.01 significant difference.
(XLSX)

S6 Table The significant SNPs of gel consistency. (XLSX)

S7 Table The significant SNPs of pericarp color in 2014 and 2015. (XLSX)
